# Record of Mortality in the City of Buffalo, for the Month of November, 1854

**Published:** 1855-01

**Authors:** J. M. Newman

**Affiliations:** Health Physician


ART. XI.—*Record of Mortality in the City of Buffalo, for the Month of November*, 1854. By J. M. NEWMAN, M. D., Health Physician.

| DISEASES. | No. | Males. | Females. |
|---|---|---|---|
| Accidens, | 2 | 1 | 1 |
| Asphyxia Immersorum, | 2 | 2 | |
| Cancer Mammæ, | 1 | | 1 |
| Cholera Asphyxia, | 2 | 2 | |
| Cynanche Trachealis, | 1 | 1 | |
| Debilitas, | 3 | | 1 |
| Diarrhœa, | 3 | 2 | 1 |
| Dysenteria, | 3 | 1 | 2 |
| Eclampsia, | 10 | 5 | 3 |
| Febris Assidua, | 1 | | 1 |
| " Scarlatina, | 12 | 5 | 5 |
| " Typhoid, | 9 | 5 | 4 |
| " Typhus, | 3 | 1 | 2 |
| Hydrops, | 5 | 3 | 2 |
| " Cerebri, | 8 | 4 | 3 |
| Hyperæmia Cerebri, | 1 | 1 | |
| Intemperantia, | 3 | 2 | 1 |
| Intussusceptio Intestinorum, | 1 | 1 | |
| Marasmus, | 1 | | |
| Morbus Brightii, | 1 | 1 | |
| Morbi Cordis, | 2 | 2 | |
| Morbi Hepaticus, | 4 | 1 | 2 |
| Morbus Spinalis, | 1 | 1 | |
| Natus Mortuus, | 6 | 1 | 2 |
| Peritonitis, | 1 | 1 | |
| Phthisis Pulmonalis, | 19 | 9 | 10 |
| Pneumonia, | 1 | 1 | |
| " Typhoides, | 1 | | 1 |
| Rubeola, | 2 | 2 | |
| Senectus, | 2 | 1 | 1 |
| Ulceratio Coli, | 1 | 1 | |
| Ignotus, | 21 | 9 | 10 |
| | 133 | | |

| | |
|---|---|
| Males, | 66 |
| Females, | 53 |
| Sex not given, | 14 |
| Total, | 133 |

REGISTER OF MORTALITY—(Continued.)

AGES.

| | | | |
|---|---|---|---|
| Still-born, | 6 | Between 6 years and 12 years, | 10 |
| 1 day, | 0 | " 12 " " 20 " | 7 |
| Between 1 day and 15 days, | 9 | " 20 " " 30 " | 17 |
| " 15 days and 30 days, | 3 | " 30 " " 40 " | 16 |
| " 1 month and 3 months, | 8 | " 40 " " 50 " | 11 |
| " 3 months and 6 months, | 5 | " 50 " " 60 " | 6 |
| " 6 " " 9 " | 2 | " 60 " " 70 " | 3 |
| " 9 " " 12 " | 4 | " 70 " " 80 " | 3 |
| " 1 year and 3 years, | 13 | " 80 " " 90 " | 0 |
| " 3 years and 6 years, | 9 | " 90 " " 100 " | 1 |
| Total, | | | 133 |

NATIVITIES.

| | | | |
|---|---|---|---|
| American, | 51 | Belgian, | 1 |
| English, | 5 | French, | 0 |
| Scotch, | 0 | Swiss, | 0 |
| Irish, | 14 | Hollander, | 0 |
| Canadian, | 3 | Colored, | 3 |
| German, | 34 | Country not given, | 22 |
| Total, | | | 133 |

